# Predictors of Premature Rupture of Membranes among Pregnant Women in Rural Uganda: A Cross-Sectional Study at a Tertiary Teaching Hospital

**DOI:** 10.1155/2020/1862786

**Published:** 2020-03-02

**Authors:** Simon Byonanuwe, Emmanuel Nzabandora, Baltazar Nyongozi, Theophilus Pius, David Santson Ayebare, Collins Atuheire, Wilson Mugizi, Martin Nduwimana, Maxwell Okello, Yarine Fajardo, Robinson Ssebuufu

**Affiliations:** ^1^Department of Obstetrics and Gynaecology, Kampala International University Teaching Hospital, Uganda; ^2^Department of Medical Laboratory Science, Kampala International University Teaching Hospital, Uganda; ^3^Interdisciplinary Research & Development Center Limited, Mbarara, Uganda; ^4^Department of Public Health, Kampala International University-Western Campus, Uganda; ^5^Postgraduate Studies & Research Directorate, Kampala International University-Western Campus, Uganda; ^6^Department of Paediatrics, Kampala International University Teaching Hospital, Uganda; ^7^Department of Surgery, Kampala International University Teaching Hospital, Uganda

## Abstract

**Background:**

Premature rupture of membranes (PROM) is a common condition in developed and developing countries and poses a serious threat to the maternal and fetal well-being if not properly managed. This study delineated the prevalence and predictors of PROM in the western part of Uganda so as to guide specific preventive measures.

**Methods:**

A cross-sectional study design was conducted in the months of September 2019 to November 2019. A total of 334 pregnant women above 28 weeks of gestation admitted at the maternity ward of KIU-TH were consecutively enrolled. Interviewer-administered questionnaires were used to obtain the data. Descriptive statistics followed by binary logistic regression were conducted. All data analyses were conducted using STATA 14.2.

**Results:**

Of the 334 pregnant women enrolled, the prevalence of PROM was found to be 13.8%. The significant independent predictors associated with lower odds of PROM were no history of urinary tract infection (UTI) in the month preceding enrollment into the study (aOR = 0.5, 95% CI: 0.22-0.69, *p* = 0.038) and gestational age of 37 weeks or more (aOR = 0.3, 95% CI: 0.14-0.71, *p* = 0.038) and gestational age of 37 weeks or more (aOR = 0.3, 95% CI: 0.14-0.71, *p* = 0.038) and gestational age of 37 weeks or more (aOR = 0.3, 95% CI: 0.14-0.71,

**Conclusions:**

Majorly urinary tract infections, low gestational age, and abortions influence premature rupture of membranes among women. There is a great need for continuous screening and prompt treatment of pregnant women for UTI especially those with history of 3 or more abortions at less than 34 weeks of gestation.

## 1. Introduction

PROM is defined as the spontaneous rupture of the fetal membranes prior to onset of labour [1]. It may occur when the fetus is 37 weeks or more of gestation (term premature rupture of membranes (TPROM)) or before 37 weeks of gestation (preterm premature rupture of membranes (PPROM)) [2]. PROM generally affects between 5 and 15% of all pregnancies worldwide with a relatively higher incidence in Africa [3–5]. About 3% of the pregnancies are complicated with preterm premature rupture of membranes [6, 7]. For several years, PROM has been the subject of several clinical and epidemiologic studies and is considered one of the great obstetrical syndromes responsible for spontaneous preterm birth and its related complications such as respiratory distress syndrome, intraventricular haemorrhage, and necrotizing enterocolitis with associated high perinatal mortality rates [8]. Up to 50% preterm births and 80% maternal clinical and subclinical infections have been associated with PROM worldwide with a fourfold increased risk of fetal mortality [9]. An estimated 65 perinatal deaths per 1000 live births following PROM have been reported in Mulago Hospital in Uganda [10]. However, since time immemorial, its aetiology has remained obscure. Nevertheless, the efforts of various scholars have continued to demonstrate the association of various obstetric and gynaecologic factors with premature rupture of membranes such as prior history of PROM or preterm labour, genital infections, prior abortions, multiple gestation, polyhydramnios, and prior cervical procedures [11–15] among others. A few available recently conducted studies in central Uganda have only attempted to describe the association of genital infections with PROM [16, 17]. There is paucity of information as regards predictors of PROM in western Uganda. This study is aimed at delineating the predictors of PROM in this area to enable early surveillance for mothers at risk during antenatal care so as to reduce associated complications and/or occurrence.

## 2. Methods

This was a cross-sectional study conducted in the months of September 2019 to November 2019 with an aim of determining the prevalence and predictors of PROM at KIU-TH. The study was conducted in the maternity ward of KIU-TH, a private nonprofit teaching hospital for Kampala International University located in Bushenyi district in southwestern Uganda about 370 kilometers from the center of Kampala city. The hospital is composed of different departments under different specialties offering both outpatient and a 700-bed capacity for the inpatients. The obstetrics and gynaecology department where this study was conducted comprises one of the departments of the hospital and operates both outpatient and inpatient services with an 85-bed capacity. The inclusion criteria were all pregnant mothers both adults and emancipated minors above 28 weeks of gestation admitted in the maternity ward that consented to participate in the study. The exclusion criteria were any pregnant women with mental illness and those who were unconscious at the time of admission. These were excluded from the study because it was not possible to interview them. Sample size was determined using Daniel's formula [18] as shown below:

(1) $$\begin{array}{c} n=\frac{{\left(z_{\alpha +}z_{\beta }\right)}^{2}p\;\left(1-p\right)}{d^{2}}, \end{array}$$

where *n* is the desired sample size, *z*_*α*_ is the *z*-statistic at *α* = 1.96 (95% level of confidence), *z*_*β*_ is the *z*-statistic at *β* = 0.84, *p* is the prevalence of PROM in Uganda (12.1%) [10], and *d* is the level of precision (0.05).

Therefore, *n* = ((1.96 + 0.84)^2^ × 0.121 (1 − 0.121))/(0.05)^2^ = 334.

A total of 548 patients were admitted on the ward during the study, of which 365 were pregnant women above 28 weeks of gestation. Out of the 365 patients, 29 opted out for the study following education and counseling about the study. Two patients, one with a mental illness and the other one who was unconscious at the time of admission, were excluded from the study as it was not possible to interview them. These continued with the usual routine assessment and management on the ward. All the other 334 pregnant women above 28 weeks of gestation were enrolled into the study and underwent a data collection process as is described subsequently.

### 2.1. Informed Consent Process

Voluntary recruitment of study participants both adults and emancipated minors was done. Informed consent from participants was obtained after fully explaining the details of the study in both the local language (Runyankore) and the national official language (English). An informed consent document both in Runyankore and English approved by the Research Ethics Committee of Kampala International University was signed by every participant, the investigator, and a witness. Participants were not forced to enroll if they did not want to. The participant was free to withdraw from the study at any time she wished, without coercion or compromise of care that she was entitled to.

### 2.2. Data Collection

Consecutive enrolment of all pregnant women who met the inclusion criteria for the study was done until the target population was realised. After counseling about the study, a written informed consent was obtained prior to enrolment as described above. Women with history suggestive of premature rupture of membranes, that is, history of leaking of clear liquor per vagina, underwent further clinical assessment to confirm the diagnosis as recommended by the Royal College of Obstetricians and Gynaecologists' Green-Top guideline number 73 [6]. Using an aseptic technique, under sufficient light, a sterile Cusco's bivalve self-retaining vaginal speculum was placed. Visualization of pooling of fluid in the posterior vaginal fornix of the patient was consistent with premature rupture of membranes in the context of this study. A structured investigator-administered pretested questionnaire about the different variables was used to obtain data. This was characterized by investigator-directed interview with the participant about the different predictors such as history of any previous preterm deliveries, abortions, and caesarean sections or any prior cervical procedures in local language (Runyankore) or in English for the participants who did not understand Runyankore. Data about the history of urinary tract infection (UTI) relied on the finding of symptoms of urinary tract infection that in the context of this study were defined by history of pain while passing urine (dysuria) and/or burning sensation while passing urine with associated lower abdominal pain in the previous one month. No laboratory assessment or any other forms of investigations were done or looked for to confirm the UTI since it was beyond the definition of this variable according to the context of the study. Meanwhile, all the study participants were recommended for and linked up with the obstetrician on duty for appropriate routine care and management based on the KIU-TH protocols.

### 2.3. Analysis

Data from questionnaires were entered into Microsoft excel version 2010 and then imported into STATA version 14.2. Data analysis and presentation was carried out according to specific objectives. Prevalence of PROM was summarized as frequencies and percentages and presented using a pie chart. Predictors of PROM were assessed using binary logistic regression. Both bivariate and multivariate logistic regression analyses were carried out. The variables in the final multivariate model were significant when *p* ≤ 0.05. The measure of association was reported as odds ratios with corresponding 95% confidence interval and *p* value. All statistical analyses were carried out in STATA version 14.2.

## 3. Results

### 3.1. Prevalence of PROM among Pregnant Women above 28 Weeks of Gestation Admitted at KIU-TH

**Figure 1:**
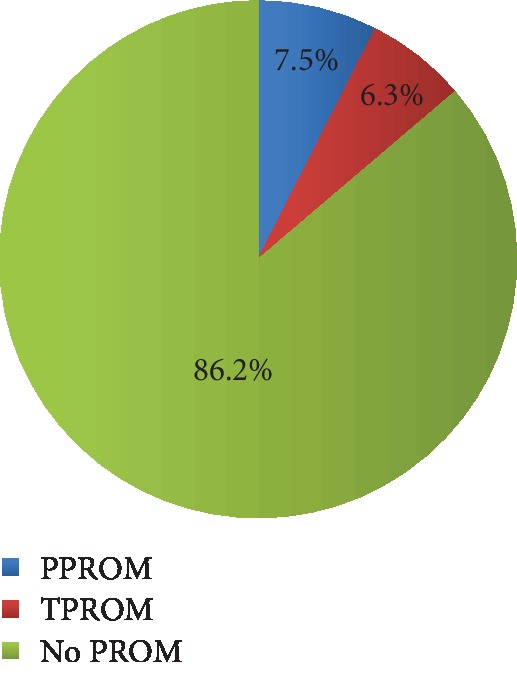
Prevalence of PROM among pregnant women admitted at KIU-TH.

Of the 334 pregnant women enrolled in the study, the overall prevalence of premature rupture of membranes was 46 (13.8%), with 25 (7.5%) participants with preterm premature rupture of membranes (PPROM) and 21 (6.3%) with term premature rupture of membranes (TPROM). However, majority did not have PROM. This is shown in Figure 1.

### 3.2. Predictors of PROM

This study found that history of urinary tract infections, number of abortions, and gestational age were the independent predictors of premature rupture of membranes among pregnant women above 28 weeks of gestation admitted at KIU-TH. Specifically, pregnant women with no history of urinary tract infections were less likely to have premature rupture of membranes (**aOR** = 0.5, 95% CI: 0.22-0.69, **p** = 0.038). Also, pregnant women of gestational age 37 weeks or more were less likely to experience premature rupture of membranes compared to their counterparts with gestational age less than 34 weeks (**aOR** = 0.3, 95% CI: 0.14-0.71, **p** = 0.01). Also, a higher number of abortions showed higher odds of premature rupture of membranes. For instance, pregnant women with three or more abortions were 13.1 times more likely to have premature rupture of membranes (**aOR** = 13.1, 95% CI: 1.12-153.62, **p** = 0.05). This is shown in Table 1.

## 4. Discussion of the Findings

The prevalence of PROM among women above 28 weeks of gestation admitted at KIU-TH was 13.8%. This prevalence of PROM lies within the worldwide range of 5 to 15% as reported by Huang et al. [3] and Shadma and Aymen [4] although it is higher than that reported by Maryuni [14] in Indonesia (10%), Abouseif et al. [19] in Egypt (4.7%), and Kayiga et al. [10] in Mulago Hospital in central Uganda (12.1%). This discrepancy was attributed to the contextual differences in the study setting. This particular study was conducted in a relatively rural setup and in the only well-equipped facility with which referral of pregnant women with PROM is made. The associated low socioeconomic status in such a rural setting could also explain the high prevalence given its known close association with PROM as suggested by Adewumi et al. [20] and Hackenhaar et al. [21]. Our prevalence was however lower than that reported in Ethiopia by Hailemariam et al. [5] where the overall prevalence of PROM was found to be 14.6%. Population differences such as the comparatively lower socioeconomic status (75.2%) of their study participants coupled with the poor antenatal care seeking behavior due to geographic hindrances are probably responsible for this discrepancy. Our prevalence was also lower than that reported by Xia et al. (2015) in East China (15.3%) probably due to the reported high prevalence of vaginal infections among their study population.

This study found a significant likelihood of PROM with number of abortions in that pregnant women with history of three or more abortions were more than 13-fold likely to have PROM (aOR = 13.1, 95% CI: 1.12-153.62, *p* = 0.05). This finding bears similarity to that of Boskabadi et al. [11] in Iran and Kaye [22] in Mulago Hospital, central Uganda, who found a significant association between two or more induced abortions with PROM. This was attributed to the fact that women with three or more abortions are likely to have a short cervix, which per se has been documented to increase the risk of PROM as suggested by Ana-Maria et al. [23]. This finding however does not coincide with that of Assefa et al. [7] in Ethiopia and Seema and Mamta [2] in India who were unable to establish any significant association between number of abortions and PROM. This discrepancy was attributed to population-specific differences as regards their perception of abortion in addition to differences in the disclosure of the number of abortions in relation to fear of associated stigma and criminalization across countries.

This study also established that history of urinary tract infections (UTIs) in the previous one month was a significant predictor of a higher likelihood of PROM. Pregnant women with no history of UTIs in the previous one month were less likely to experience PROM compared to those with prior history of urinary tract infections (aOR = 0.5, 95% CI: 0.22-0.69, *p* = 0.038). Although this finding is contrary to findings by Hackenhaar et al. [21] in Brazil, similar observations have been reported by other researchers such as Patil and Patil [24] in India, Gahwagi et al. [13] in Libya, and Boskabadi et al. [25] in Iran who found UTIs to be the major factors associated with PROM. UTIs are potential reservoirs for bacteria that cross to the vagina and ascend through the cervical canal to the membranes where they cause localized inflammation. The bacteria produce a number of proteolytic enzymes such as collagenase and gelatinase that can cause local weakening of the membranes. Also, the subsequent prostaglandin production resulting from localized inflammation leads to occult contractions with increased shearing stress at the internal cervical os resulting into PROM.

Although studies by Gahwagi et al. [13] in Libya and Boskabadi and Zakerihamidi [25] in Iran showed no significant association between gestational age with PROM, our study found that pregnant women of 37 weeks and more were less likely to experience PROM compared to early gestation women of less than 34 weeks (aOR = 0.3, 95% CI: 0.14-0.71, *p* = 0.01). Similar observations were made by Shadma and Aymen [4] in India, Abouseif et al. [19] in Egypt, and Msomi et al. [26] in South Africa where majority of the women with PROM were below 34 weeks of gestation. This is probably due to gestational age-related differences regarding fetal presentation, fetal position, and fetal lie, as well as maternal physiology. Pregnant women below 34 weeks of gestation are likely to have fetal malpresentation, fetal malposition, and abnormal fetal lie compared with those at 37 weeks or more, as the fetus has enough freedom of movement until the later months of pregnancy when it becomes relatively fixed as suggested by Dutta [27]. In addition, these women are at higher risk of urinary tract infections compared to those at 37 weeks of gestation or more partly due to the relatively high progesterone hormone level-associated hydronephrosis and urinary stasis as suggested by Gary et al. [28]. As Xia et al. [29] in China, Patil and Patil [24], and Seema and Mamta [2] in India observed, these factors are noted to contribute to the risk of premature rupture of membranes.

### 4.1. Study Strengths

This is the first documented study conducted in a rural western Uganda to report the prevalence and predictors of premature rupture of membranes. Also, our sample size was relatively high which improved the precision of the study.

### 4.2. Study Limitations

We could not establish causal relationship given that this was a cross-sectional study.

## 5. Conclusions

The prevalence of premature rupture of membranes at Kampala International University Teaching Hospital is high compared to the national average. History of urinary tract infection, number of abortions, and gestational age are the major obstetric and gynaecologic predictors of premature rupture of membranes at KIU-TH. Vigilance must be ensured by health care workers at KIU-TH as regards screening for urinary tract infections and timely initiation of treatment for all mothers with UTIs during antenatal care. Women with history of recurrent abortion need to be sensitized by all the attending health care workers on risk of PROM and advised on the need for close monitoring during their subsequent pregnancies.

## Figures and Tables

**Table 1:** Predictors of PROM at KIU-TH (*N* = 334).

		PROM		UOR (95% CI)	*p*	aOR (95% CI)	*p*
		Yes	No				
Gravidity	<2	18 (17.1)	87 (82.9)	1.0			
	2-4	20 (11.2)	159 (88.8)	0.6 (0.31-1.21)	0.266		
	5+	8 (16.0)	42 (84.0)	0.9 (0.37-2.29)	0.957		

Parity	<2	28 (14.6)	164 (85.4)	1.0			
	2-4	14 (11.6)	107 (88.4)	0.8 (0.39-1.52)	0.447		
	5+	4 (19.1)	17 (80.9)	1.4 (0.43-5.00)	0.588		

Gestational age (weeks)	<34	11 (23.4)	36 (76.6)	1.0			
	34-36	14 (28.0)	36 (72.0)	1.3 (0.51-3.18)	0.605	1.2 (0.47-3.07)	0.66
	37+	21 (8.9)	216 (91.1)	0.3 (0.14-0.72)	0.006^∗^	0.3 (0.14-0.71)^∗^	0.01^∗^

Number of ANC	<4 times	12 (13.5)	71 (85.5)	1.0			
	4 or more	34 (14.5)	217 (86.5)	1.1 (0.53-2.20)	0.834		

History of preterm labour	Yes	4 (14.8)	23 (85.2)	1.0			
	No	42 (13.7)	265 (86.3)	1.0 (0.41-2.33)	0.197		

History of caesarian section	Yes	8 (12.70)	55 (87.30)	1.0			
	No	38 (14.02)	233 (85.98)	1.1 (0.50-2.54)	0.784		

History of UTI	Yes	11 (23.91)	35 (76.09)	1.0			
	No	35 (12.15)	253 (87.85)	0.4 (0.21-0.95)	0.035^∗^	0.5 (0.22-0.69)	0.038^∗^

Number of abortions	None	39 (13.73)	245 (86.27)	1.0			
	1-2	5 (10.64)	42 (89.36)	0.7	0.564	0.7 (0.26-1.94)	0.473
	3+	2 (66.67)	1 (33.33)	12.6 (1.11-141.88)	0.041^∗^	13.1 (1.12-153.62)	0.05^∗^

History of surgical evacuation	Yes	2 (8.33)	22 (91.67)	1.0	0.429		
	No	44 (14.19)	266 (85.81)	1.8 (0.41-8.01)			

Cervical procedures	Yes	2 (40.00)	3 (60.00)	1.0			
	No	44 (13.37)	285 (86.63)	0.2 (0.04-1.43)	0.115		

^∗^ *p* ≤ 0.05. UOR = unadjusted odds ratio; aOR = adjusted odds ratio; CI = confidence interval; *p* = significance level.

## Data Availability

The data that were used to obtain the findings are available from the corresponding author if needed.

## Abbreviations

KIU-TH: Kampala International University Teaching Hospital
PROM: Premature rupture of membranes
UTI: Urinary tract infections.
